# Extracellular clusterin limits the uptake of α‐synuclein fibrils by murine and human astrocytes

**DOI:** 10.1002/glia.23920

**Published:** 2020-10-12

**Authors:** Alice Filippini, Veronica Mutti, Gaia Faustini, Francesca Longhena, Ileana Ramazzina, Federica Rizzi, Alice Kaganovich, Dorien A. Roosen, Natalie Landeck, Megan Duffy, Isabella Tessari, Federica Bono, Chiara Fiorentini, Elisa Greggio, Luigi Bubacco, Arianna Bellucci, Mariacristina Missale, Mark R. Cookson, Massimo Gennarelli, Isabella Russo

**Affiliations:** ^1^ Unit of Biology and Genetics, Department of Molecular and Translational Medicine University of Brescia Brescia Italy; ^2^ Unit of Pharmacology, Department of Molecular and Translational Medicine University of Brescia Brescia Italy; ^3^ Department of Medicine and Surgery University of Parma Parma Italy; ^4^ Laboratory of Neurogenetics National Institute on Aging, National Institutes of Health Bethesda Maryland USA; ^5^ Department of Biology University of Padova Padova Italy; ^6^ Laboratory of Personalized and Preventive Medicine University of Brescia Brescia Italy; ^7^ Genetics Unit IRCCS Istituto Centro S. Giovanni di Dio Fatebenefratelli Brescia Italy; ^8^ Present address: Genetics Unit IRCCS Istituto Centro S. Giovanni di Dio Fatebenefratelli Brescia Italy

**Keywords:** α‐synuclein, astrocytes, clusterin, hiPSC, Parkinson's disease

## Abstract

The progressive neuropathological damage seen in Parkinson's disease (PD) is thought to be related to the spreading of aggregated forms of α‐synuclein. Clearance of extracellular α‐synuclein released by degenerating neurons may be therefore a key mechanism to control the concentration of α‐synuclein in the extracellular space. Several molecular chaperones control misfolded protein accumulation in the extracellular compartment. Among these, clusterin, a glycoprotein associated with Alzheimer's disease, binds α‐synuclein aggregated species and is present in Lewy bodies, intraneuronal aggregates mainly composed by fibrillary α‐synuclein. In this study, using murine primary astrocytes with clusterin genetic deletion, human‐induced pluripotent stem cell (iPSC)‐derived astrocytes with clusterin silencing and two animal models relevant for PD we explore how clusterin affects the clearance of α‐synuclein aggregates by astrocytes. Our findings showed that astrocytes take up α‐synuclein preformed fibrils (pffs) through dynamin‐dependent endocytosis and that clusterin levels are modulated in the culture media of cells upon α‐synuclein pffs exposure. Specifically, we found that clusterin interacts with α‐synuclein pffs in the extracellular compartment and the clusterin/α‐synuclein complex can be internalized by astrocytes. Mechanistically, using clusterin knock‐out primary astrocytes and clusterin knock‐down hiPSC‐derived astrocytes we observed that clusterin limits the uptake of α‐synuclein pffs by cells. Interestingly, we detected increased levels of clusterin in the adeno‐associated virus‐ and the α‐synuclein pffs‐ injected mouse model, suggesting a crucial role of this chaperone in the pathogenesis of PD. Overall, our observations indicate that clusterin can limit the uptake of extracellular α‐synuclein aggregates by astrocytes and, hence, contribute to the spreading of Parkinson pathology.

## Main Points

- Extracellular clusterin binds α‐synuclein fibrils thus limiting their uptake by murine and human astrocytes.
- Reduced levels of clusterin improve α‐synuclein aggregates clearance by astrocytes.

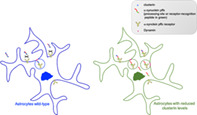

## 1 INTRODUCTION

Parkinson's disease (PD) is a common neurodegenerative disorder pathologically characterized by neuronal cell death in many brain regions and the presence of protein inclusions, Lewy bodies, and Lewy neurites, in surviving neurons (Del Tredici & Braak, 2016; Forno, 1996; Lees, Hardy, & Revesz, 2009). These protein inclusions are mainly composed of aggregated forms of the normally presynaptic neuronal protein, α‐synuclein (Spillantini et al., 1997). Accumulating evidence suggests that α‐synuclein aggregates can be secreted from neurons during cell stress and/or degeneration, leading to a higher concentration and further aggregation of α‐synuclein in the extracellular space, which could initiate or propagate the spreading of toxic forms of α‐synuclein between neurons (Bieri, Gitler, & Brahic, 2018; Booth, Hirst, & Wade‐Martins, 2017; Lopes da Fonseca, Villar‐Piqué, & Outeiro, 2015). Therefore, approaches that can improve the uptake/clearance of extracellular α‐synuclein aggregates might be beneficial to prevent cell‐to‐cell α‐synuclein toxic forms propagation and the progression of the pathology.

Although all major brain cell types are able to uptake disease‐related protein aggregates, glial cells appear to be the most efficient scavengers (Filippini, Gennarelli, & Russo, 2019; Jung & Chung, 2018). Interestingly, astrocytes have been identified as key players in the clearance of extracellular α‐synuclein. Toxic species of α‐synuclein can be efficiently internalized by astrocytes and degraded through the endo‐lysosomal pathway (Lindström et al., 2017; Loria et al., 2017; Sacino et al., 2017). However, upon extensive endocytosis of α‐synuclein aggregates, astrocytes may develop large intracellular deposits, as protein degradation capacity becomes saturated (Lindström et al., 2017; Sacino et al., 2017). Consistent with this hypothesis, α‐synuclein‐containing inclusions occur in astrocytes of PD cases postmortem (Braak, Sastre, & Del Tredici, 2007; Wakabayashi, Hayashi, Yoshimoto, Kudo, & Takahashi, 2000). As astrocytes do not natively express α‐synuclein (Aflaki et al., 2020), this might indicate that aggregated species are released from affected neurons and then taken up by astrocytes to limit the spread of neuropathology.

As well as uptake via endocytosis, cells may also detoxify extracellular misfolded or aggregated proteins through molecular chaperones (Hartl, Bracher, & Hayer‐Hartl, 2011; Yerbury, Stewart, Wyatt, & Wilson, 2005). Of these, clusterin was the first to be identified and to be proposed as part of the extracellular protein quality control system (Wilson & Easterbrook‐Smith, 2000; Yerbury et al., 2005). It has been shown that clusterin influences the aggregation state of several client proteins, including α‐synuclein, and is able to interact with intermediate aggregated species during the course of fibrillation (Yerbury et al., 2007). Additionally, clusterin has been reported to affect beta‐amyloid endocytosis depending on the protein conformation state and/or the cell type investigated (Hammad, Ranganathan, Loukinova, Twal, & Argraves, 1997; Mulder, Nielsen, Blankenstein, Eikelenboom, & Veerhuis, 2014; Nielsen et al., 2010; Zlokovic et al., 1996). Therefore, clusterin may function in both extracellular refolding and endocytosis and may be crucial for the extracellular proteins that are prone to aggregation. Clusterin is predominantly expressed in the central nervous system by astrocytes (Foster, Dangla‐Valls, Lovestone, Ribe, & Buckley, 2019; Morgan et al., 1995; Pasinetti, Johnson, Oda, Rozovsky, & Finch, 1994), thus suggesting a non‐cell autonomous role of this chaperone in the pathogenesis of neurodegenerative diseases.

Here, we explored the role of clusterin on the clearance of α‐synuclein aggregates by astrocytes. We find that clusterin levels are modulated in astrocytes, both in primary murine and in human‐induced pluripotent stem cell (iPSC)‐derived astrocytes (hiAstrocytes), upon exposure to preformed fibrils (pffs) of α‐synuclein and in two different PD models in vivo. Specifically, we show that astrocytes internalize α‐synuclein pffs through dynamin‐dependent endocytosis and that extracellular clusterin levels are diminished in the culture media of cells treated with pffs, suggesting that clusterin is endocytosed with pffs. Mechanistically, using clusterin knock‐out (KO) primary astrocytes and clusterin knock‐down (KD) hiAstrocytes, we report that clusterin acts at the level of endocytosis to limit the uptake of α‐synuclein pffs.

Overall, our findings indicate that extracellular clusterin by binding to α‐synuclein pffs limits their endocytosis by astrocytes and, importantly, suggest that this chaperone may contribute to the spreading of α‐synuclein pathology in PD.

## 2 MATERIALS AND METHODS

### 2.1 Animals and primary astrocytes

Animals were maintained under a 12 hr light–dark cycle at room temperature (RT) of 22°C and had ad libitum food and water. C57BL/6 clusterin wild‐type (WT) and clusterin KO mice were housed at the Animal Facility of Parma, University of Parma (ID: 2015/120601 and 09/2018‐UT). C57BL/6J mice were housed at the University of Brescia. Animal procedures were performed in accordance with European Community Directive 2010/63/UE and approved by the Ethics Committee of the University of Brescia (Project ID: 800‐2017‐PR).

Primary astrocytic cultures were derived from postnatal Days 2–4 (P2‐P4) C57BL/6J WT mice or C57BL/6 clusterin WT or KO mice. Cerebral cortices were dissociated in cold PBS, the cell suspension was then allowed to settle for 5 min at RT and the top fraction was collected and centrifuged for 5 min at 1,000 rpm. Subsequently, the cells were resuspended in complete astrocytes medium containing 50% DMEM high glucose (ThermoFisher Scientific), 50% Ham's F12 supplement (ThermoFisher Scientific), 10% fetal bovine serum (FBS, ThermoFisher Scientific), 2 mM Sodium Pyruvate (ThermoFisher Scientific), 2 mM l‐glutamine (ThermoFisher Scientific), and Penicillin/Streptomycin (ThermoFisher Scientific). Cell suspension obtained from five brains was plated on T175 flasks and maintained in culture at 37°C with 5% CO_2_. After 4 days, the medium was changed, and the culture was maintained until Day 10 when the cells were subcultured for experimental applications.

### 2.2 Generation and differentiation of hiAstrocytes

Human iPSCs were obtained from fibroblasts of a healthy donor and characterized as previously described (Bono et al., 2018). Informed consent was obtained from the donor prior to cell donation, which was previously approved by the local ethics committee (CEIOC—“San Giovanni di Dio Fatebenefratelli Hospital, Brescia, Italy, 44/2001 and 39/2005).

Human iPSCs were differentiated into astrocytes following a protocol consisting of neural stem cell induction, neural stem cell expansion and astrocytes terminal differentiation steps (Yan et al., 2013). For neuronal induction, iPSCs were plated on Matrigel (BD Biosciences) and cultured for 24 hr in Stemflex medium (Gibco, Life Technologies), followed by incubation with PSC Neural Induction Medium (Gibco, Life Technologies). After 7 days, primitive neural stem cells were dissociated with Accutase (Life Technologies) and cultured with Neural Expansion Medium containing 50% Neurobasal Medium (Gibco, Life Technologies), 50% advanced DMEM/F12 (Gibco, Life Technologies) supplemented with Neural Induction Supplement (Gibco, Life Technologies). Cells were expanded in the presence of the Y27632 ROCK inhibitor (10 μM; Tocris) for preventing cell death. For astrocyte differentiation, cells were dissociated using Accutase and cultured in DMEM (Euroclone) supplemented with N2 (Life Technologies) and 1% FBS (Sigma Aldrich); hiAstrocytes were used for all the experimental applications between Days 25 and 40 of differentiation.

### 2.3 Production of α‐synuclein pffs and cell treatment

Human α‐synuclein pffs were generated from recombinant α‐synuclein produced by a lipid A mutant of *Escherichia coli*, BL21(DE3) that has strongly reduced endotoxin production. Specifically, after purification, α‐synuclein was incubated for 15 days at 37°C under constant shaking to induce aggregation. Enriched‐pffs, isolated by the soluble part of the preparation by centrifugation at 14,000 rpm for 15 min, was then quantified relative to initial concentration of monomer before fibrillation, as previously described (Russo et al., 2015, 2019), and resuspended in PBS. The fibrillization of pffs was verified by Thioflavin T (ThT) and sedimentation assays (Supplementary Figure S2a,b). For ThT test, 7 μg of α‐synuclein pffs and monomer were added to 5 μM of ThT in PBS, mixed well and incubated for 15 min at RT. Control measurement was performed with 5 μM ThT in PBS for detection of background fluorescence intensity. Fluorescence emission spectra were recorded at 482 nm with excitation at 440 nm.

Primary astrocytes and hiAstrocytes were treated with α‐synuclein pffs at 10 μM or α‐synuclein monomer (M) at 10 nM as control. Considering that one fibril is estimated to contain ~10,000 monomers (van Gestel & de Leeuw, 2006), 10 nM of monomeric α‐synuclein corresponds to a~10× molar excess of α‐synuclein fibrils. In the experiments with chloroquine (CQ) and in immunofluorescence analysis, we used α‐synuclein pffs at 2 μM given that the combination of 10 μM α‐synuclein pffs with CQ‐induced cell death and the use of 10 μM α‐synuclein pffs caused a higher α‐synuclein fluorescence background generated by pffs nonspecific binding to coated surface and cell membranes. During treatment, primary astrocytes were cultured in medium containing 1% FBS.

### 2.4 pH rodo α‐synuclein pffs treatment and quantification

pH rodo α‐synuclein pffs (*α‐synuclein pffs) were generated by using pH rodoTM iFL Green Microscale Protein Labeling kit (ThermoFisher Scientific) following manufacturer's instructions. In these experiments *α‐synuclein pffs were used at 2 μM to avoid saturation of fluorescence imaging by confocal microscopy. Primary astrocytes were treated with *α‐synuclein pffs for 4 hr, while hiAstrocytes were treated for 48 hr. This difference of timing is based on the different ability of the two astrocytic cell types to uptake α‐synuclein pffs. After treatment with *α‐synuclein pffs, the cells were fixed in 4% PFA for 15 min at RT and mounted using Prolong Gold Antifade reagent with DAPI (ThermoFisher Scientific). Quantification of intracellular *α‐synuclein pffs was performed using ImageJ software, calculated as fluorescence intensity divided by cellular area and expressed as fluorescence intensity/μm^2^. At least 50 cells were randomly chosen in a minimum of three independent experiments. Images were acquired with a Zeiss LSM 510 confocal microscope using Zeiss 63×/1.4 numerical aperture oil‐immersion objective (Carl Zeiss AG).

### 2.5 Compound treatments

The dynamin‐inhibitor Dynasore, dissolved in DMSO, was used at 80 μM. Primary astrocytes were incubated with Dynasore 30 min before treatment with *α‐synuclein pffs and then kept in culture for another 4 hr before fixation. CQ was used at 25 μM. Primary astrocytes were incubated for 30 min before treatment with α‐synuclein pffs and then maintained in culture for another 16 hr before cells lysis.

### 2.6 Cell lysis and western blotting

Primary astrocytes and hiAstrocytes were washed twice with PBS, solubilized with lysis buffer (20 mM Tris–HCl pH 7.5, 150 mM NaCl, 1 mM EDTA, 2.5 mM sodium pyrophosphate, 1 mM β‐glycerophosphate, 1 mM Na_3_VO_4_, 1% Triton‐X‐100) supplemented with protease inhibitor cocktail (Sigma‐Aldrich), incubated in ice for 30 min and cleared by centrifugation 14,000 rpm at 4°C.

Total proteins were then separated by electrophoresis on 7.5% sodium dodecyl sulfate (SDS)‐PAGE gels, while media samples were separated on 7.5% urea‐PAGE gels. Subsequently, the proteins were transferred on PVDF membrane (Bio‐Rad). After saturation with 5% nonfat dry milk, membranes were incubated 1 hr at RT with the following primary antibodies: anti‐GAPDH (ThermoFisher Scientific MA5‐15738, 1:8,000), anti‐α‐synuclein (Abcam, ab138501 MJFR 1:10,000), anti‐clusterin (mouse reactivity; R&D Systems AF2747, 1:1,000), anti‐clusterin (human reactivity; R&D Systems AF2937, 1:1,000), anti‐Glial Fibrillar Acidic Protein (GFAP, GeneTex GTX108711, 1:2,000), anti‐Excitatory Amino Acid Transporter 2 (EAAT2, Novus Biological NBP1‐20136, 1:500). Subsequently, membranes were incubated 1 hr at RT with HRP conjugated secondary antibodies (Sigma Aldrich) and finally with ECL western blot substrate (GE Healthcare).

### 2.7 Cell immunofluorescence and confocal imaging

Cells were washed once with PBS and fixed using 4% PFA for 15 min. Following three washes with PBS, cells were permeabilized with 0.3%Triton X‐100 in PBS for 5 min at RT and then saturated with blocking solution containing 5% FBS and 0.3% Triton X‐100 in PBS for 30 min at RT. Primary antibodies were diluted in blocking solution. We used the following primary antibodies: anti‐α‐synuclein 1:500 (Abcam, ab138501 MJFR), anti‐Early Endosome Antigen 1 (EEA1) 1:500 (R&D Systems, AF8047), anti‐pan cadherin 1:100 (Abcam, ab6528), anti‐clusterin 1:100 (R&D Systems AF2747), anti‐GFAP 1:200 (Dako, Z0334); anti‐S100β 1:100 (Abcam, ab868), anti‐TubβIII 1:100 (Sigma‐Aldrich, T8660). After several washes with PBS, the cells were incubated 1 hr at RT with secondary antibodies AlexaFluor 488, AlexaFluor 647, and AlexaFluor 555 (dilution 1:500; Life Technologies), then washed three times with PBS and mounted using Prolong Gold Antifade reagent containing DAPI. Images were acquired with a Zeiss LSM 510 confocal microscope using Zeiss 63×/1.4 numerical aperture oil‐immersion objective (Carl Zeiss AG).

### 2.8 Isolation of α‐synuclein pffs from cell culture medium

Cell culture media were collected after treatment with α‐synuclein pffs and centrifuged at 14,000 rpm for 20 min at 4°C. After centrifugation, the supernatant with soluble materials was discarded while the pellet containing α‐synuclein pffs was resuspended in culture medium, loaded in a 7.5% urea‐PAGE gels and subjected to western blotting analysis.

### 2.9 Co‐immunoprecipitation assay

The media collected after α‐synuclein pffs treatment were incubated with 15 μl of Dynabeads Protein G (ThermoFisher Scientific, 10007D) and 1 μg of anti‐clusterin (R&D Systems AF2747) 2 hr at 4°C on a rotating wheel. As negative control (NC), the media were incubated with 15 μl of Dynabeads Protein G. After several washes with co‐immunoprecipitation (co‐IP) lysis buffer (50 mM Tris–HCl pH 7.5, 1 mM EDTA, 0.27 M sucrose, 2.5 mM sodium pyrophosphate, 1 mM β‐glycerophosphate, 1 mM Na_3_VO_4_, 1% Triton‐X‐100 and protease inhibitors), the immune complex was resuspended in sample buffer and then subjected to immunoblotting analysis.

### 2.10 Calcium imaging

HiAstrocytes were placed in an eight‐well chamber slide (Ibidi chambers) and after 4 days of culture were incubated with Fluo4‐AM fluorophore for 1 hr at 37°C. Cells were then imaged for 30 min using a Zeiss LSM 880 confocal microscope equipped with Plan‐Apochromat 63×/1.4 numerical aperture oil‐objective. Recorded data were examined using Zen 2.3 Software (Carl Zeiss AG).

### 2.11 Cocultures of hiAstrocytes and murine midbrain neurons

HiAstrocytes were plated on Matrigel and cultured for 7 days in DMEM containing 10% FBS (Sigma Aldrich), 2 mM l‐glutamine (Sigma Aldrich) and Penicillin–Streptomycin (Sigma Aldrich). Primary midbrain neurons generated as previously described (Bono et al., 2019) were seeded on a confluent monolayer of hiAstrocytes or on Poly‐d‐lysine/laminin‐coated wells and cultured in Neurobasal medium supplemented with 2 mM l‐glutamine, 2% B27 (Gibco, Life Technologies) and Penicillin–Streptomycin for additional 7 days. Cells were then fixed using 4% PFA for 10 min at RT, saturated in PBS containing 0.1%Triton x‐100 (Sigma Aldrich) and 5% bovine serum albumin (BSA) and incubated for 16 hr with anti‐TH primary antibody (Santa Cruz, SC14007 1:500) at 4°C. After several washes, cells were incubated with the biotinylated anti‐rabbit antibody (Jackson Immunoresearch, BA‐1000 1:700) for 30 min at RT followed by incubation with avidin‐biotin horseradish peroxidase complex (Vector). Staining with peroxidase was performed by incubation of cells in PBS containing 1% 3–3′ diaminobenzidine and 0.01% H_2_O_2_ (Sigma‐Aldrich). Digital images were acquired with an Olympus IX51 microscope connected to an Olympus digital camera and morphometric measurements were performed using ImageJ software. The maximal dendrite length, the number of primary dendrites and the soma area were used as indicators of morphological parameters as previously described (Bono et al., 2018). At least 60 cells were analyzed in three independent experiments.

### 2.12 SiRNA transfection and pH rodo α‐synuclein pffs treatment

HiAstrocytes were seeded at 80% of confluence and the day after were transfected using Lipofectamine 2000 (Life Technologies) with a scrambled sequence NC (Integrated DNA Technologies; 160 nM) or with clusterin siRNA (Ambion; 160 nM; sequence 5′‐GCAGCAGAGUCUUCAUCAU‐3′), both widely used as previously reported (Bettuzzi et al., 2009; Bonacini et al., 2015; Chayka et al., 2009). After 24 hr of transfection, the cells were treated with 2 μM *α‐synuclein pffs for 24 hr and then fixed; *α‐synuclein pffs internalization was quantified as described above.

### 2.13 Animals stereotaxic surgery, immunostaining, brain lysis, and western blotting

All experimental and surgical procedures conformed to the National Research Guide for the Care and Use of Laboratory Animals were approved by the Animal Research Committees of the University of Brescia (Project ID: 719/2015‐PR) for animals housed at University of Brescia (adeno‐associated virus [AAV]‐based mouse model) and by the Institutional Animal Care and Use Committees of the US National Institute on Aging for the animals housed at NIH (α‐synuclein pffs‐based mouse model; Project ID: 463‐LNG‐2021). All achievements were made to minimize animal suffering and to reduce the number of animals used.

The plasmids for the production of AAV serotype 2/6 inducing the overexpression of human WT α‐synuclein (AAV2/6‐hα‐synuclein), driven by the Synapsin I promoter and enhanced using a woodchuck hepatitis virus posttranscriptional regulatory element, were produced as previously described (Decressac, Mattsson, Lundblad, Weikop, & Björklund, 2012; Lundblad, Decressac, Mattsson, & Björklund, 2012) and acquired by the University of Lund, Sweden, at the titers 4.7 × 10^14^ genome copies/ml. Two‐months‐old WT mice were injected in the left substantia nigra with AAV2/6‐hα‐synuclein diluted at 5 × 10^13^ genome copies/ml. Briefly, animals were anesthetized and placed in a stereotactic head frame (Stoelting). After making a midline incision of the scalp, a hole was drilled in the appropriate location for the substantia nigra at the left side of the skull. Two microliters of viral vector were injected at a rate of 0.2 μl/min with a 33‐gauge needle on a 10 μl Hamilton syringe at the following coordinates: anteroposterior −3.60; mediolateral +1.15; and dorsal–ventral −3.75 relative to bregma (Fu et al., 2012). The needle was left in place for an additional 5 min before being slowly retracted from the brain. Mice were then sacrificed at 8 weeks after the injection for immunohistochemical or biochemical analysis.

For the α‐synuclein pffs‐based mouse model, α‐synuclein pffs were generated as recently described (Patterson et al., 2019). Briefly, mouse α‐synuclein monomers (obtained from the MJFF) were diluted to 5 mg/ml and incubated for 7 days at 37°C on a shaker at 1,000 rpm. Afterward, ThT and sedimentation assay were performed for quality control (Supplementary Figure S2c,d). Prior to surgery, a fresh aliquot of α‐synuclein pffs was diluted to 2.5 μg/μl with sterile PBS and sonicated with a probe sonicator set to 30% amplitude, 60 pulses, 1 s on, 1 s off for 2 min in total.

Twelve‐months‐old mice were initially anesthetized by 5% isoflurane and kept under anesthesia using 1–2% isoflurane. Mice were placed into a stereotaxic frame where an incision was made above the midline and the skull was exposed using cotton tips. At anteroposterior +0.2 mm, mediolateral ±2.0 mm from bregma (bilateral injection), a hole was drilled into the skull and the last thin layer was removed using forceps to not damage the dura. A pulled glass capillary (blunt) attached to a 5 μl Hamilton syringe was used for injection. First, an air bubble of 1 μl was pulled in followed by 2 μl of 2.5 μg/μl of mouse α‐synuclein pffs solution (5ug total). The capillary was lowered to dorsoventral −3.2 mm from bregma into the dorsal striatum. The solution was delivered at a rate of 0.1 μl per 10 s. After the injection, the capillary was held in place for 2 min, retracted 0.1 μm, and another 1 min was waited before it was slowly withdrawn from the brain.

For the immunostaining on the AAV‐based mouse model, 8 weeks after surgery mice were anesthetized by intraperitoneal injection of chloral hydrate (400 mg/kg; Sigma‐Aldrich) and were perfused transcardially by using a 4% PFA Immunofix solution (Bio‐Optica). After 4 hr of postfixation, brains were incubated in a solution of PBS with high salt concentration (NaOH 200 mM, NaH_2_PO_4_ 245 mM, NaCl 0.9%) containing 18% sucrose for at least 24 hr, then 30 μm coronal sections were cut with a cryostat (Leica Biosystems) and conserved in 60% glycerol until used for immunostaining analysis. After permeabilization in 20% methanol and 0.3% Triton X‐100 in PBS 0.1 M, free floating slices were incubated for 1 hr at RT in blocking solution (2% vol/vol normal goat serum, 3% wt/vol BSA, 0.3% Triton X‐100 in PBS 0.1 M), and then with the primary antibody diluted in blocking solution overnight at 4°C. The following day, slices were washed with 0.3% Triton X‐100 in PBS 0.1 M and incubated with the fluorochrome‐conjugated secondary antibody in 0.3% Triton X‐100 PBS 0.1 M plus 1 mg/ml BSA for 1 hr at RT. After three washes in 0.3% Triton X‐100 in PBS 0.1 M, slices were incubated for 2 hr at RT with the second primary antibody, followed by incubation for 1 hr at RT with the optimal secondary antibody. Then, slices were mounted onto glass slides using Vectashield (Vector Laboratories) and analyzed by a Zeiss LSM 880 confocal microscope equipped with Plan‐Apochromat 63×/1.4 numerical aperture oil‐objective.

For the immunostaining on α‐synuclein pffs‐based mouse model, 4 weeks after surgery animals were perfused transcardially by using a 4% PFA. After 2 days of postfixation, brains were transferred to 30% sucrose solution and sectioning was started once brains had sunk to the bottom. The brains were then cut into 30 μm thick coronal sections and stored in antifreeze solution (0.5 M phosphate buffer, 30% glycerol, 30% ethylene glycol) at −20°C until used for immunostaining analysis. For immunostaining, sections were washed with PBS and incubated for 30 min in blocking buffer (10% NDS, 1% BSA, 0.3% Triton in PBS). Afterward, primary antibodies anti‐clusterin (R&D Systems, AF2747) and anti‐S100β (Abcam, ab52642) were used at 1:500 and incubated ON at 4°C in 1% NDS, 1% BSA, 0.3% Triton in PBS. Next day, sections were washed ×3 for 10 min each with PBS and incubated with AlexaFluor conjugated secondary antibodies for 1 hr at RT. After three washes with PBS, sections were mounted on glass slides, coverslipped using Prolong Gold Antifade mounting media (Invitrogen) and imaged using a Zeiss LSM 880 confocal microscope equipped with a Plan‐Apochromat 63×/1.4 numerical aperture oil‐objective (Carl Zeiss AG).

For western blotting analysis, fresh frozen tissues from the ventral midbrain were collected from mouse brains after cervical dislocation. Total proteins were extracted with radiommunoprepitation assay buffer made up by 50 mM Tris–HCl pH 7.4, 150 mM NaCl, NP‐40 1%, sodium deoxycholate 0.1%, SDS 0.1%, 1 mM NaF, 1 mM NaVO_4_ plus complete protease inhibitor mixture (Roche Diagnostics). Total protein concentration was measured by using the Bio‐Rad DCTM protein assay kit (Bio‐Rad Laboratories). Then, 15 μg of total proteins were separated by electrophoresis on 7.5% polyacrylamide gels and then transferred on PVDF membrane (Bio‐Rad). After saturation with 5% nonfat dry milk, membranes were incubated 1 hr at RT with anti‐GAPDH (ThermoFisher Scientific MA5‐15738, 1:8,000) and anti‐clusterin (R&D Systems AF2747, 1:1,000). Subsequently, membranes were incubated 1 hr at RT with HRP conjugated secondary antibodies (Sigma Aldrich) and finally with ECL western blot substrate (GE Healthcare).

### 2.14 Statistical analysis

All quantitative data are expressed as mean ± *SD* and represent at least three independent sets of experiments. Statistical significance of differences between two groups was assessed by unpaired *t* test or one‐sample *t* test, while for multiple comparisons by one‐way analysis of variance (ANOVA) with Tukey's post hoc test. Data were analyzed using Prism (GraphPad) and statistical significance was taken at *p* < .05.

## 3 RESULTS

### 3.1 Primary astrocytes uptake α‐synuclein pffs through dynamin‐dependent endocytosis

**FIGURE 1:**
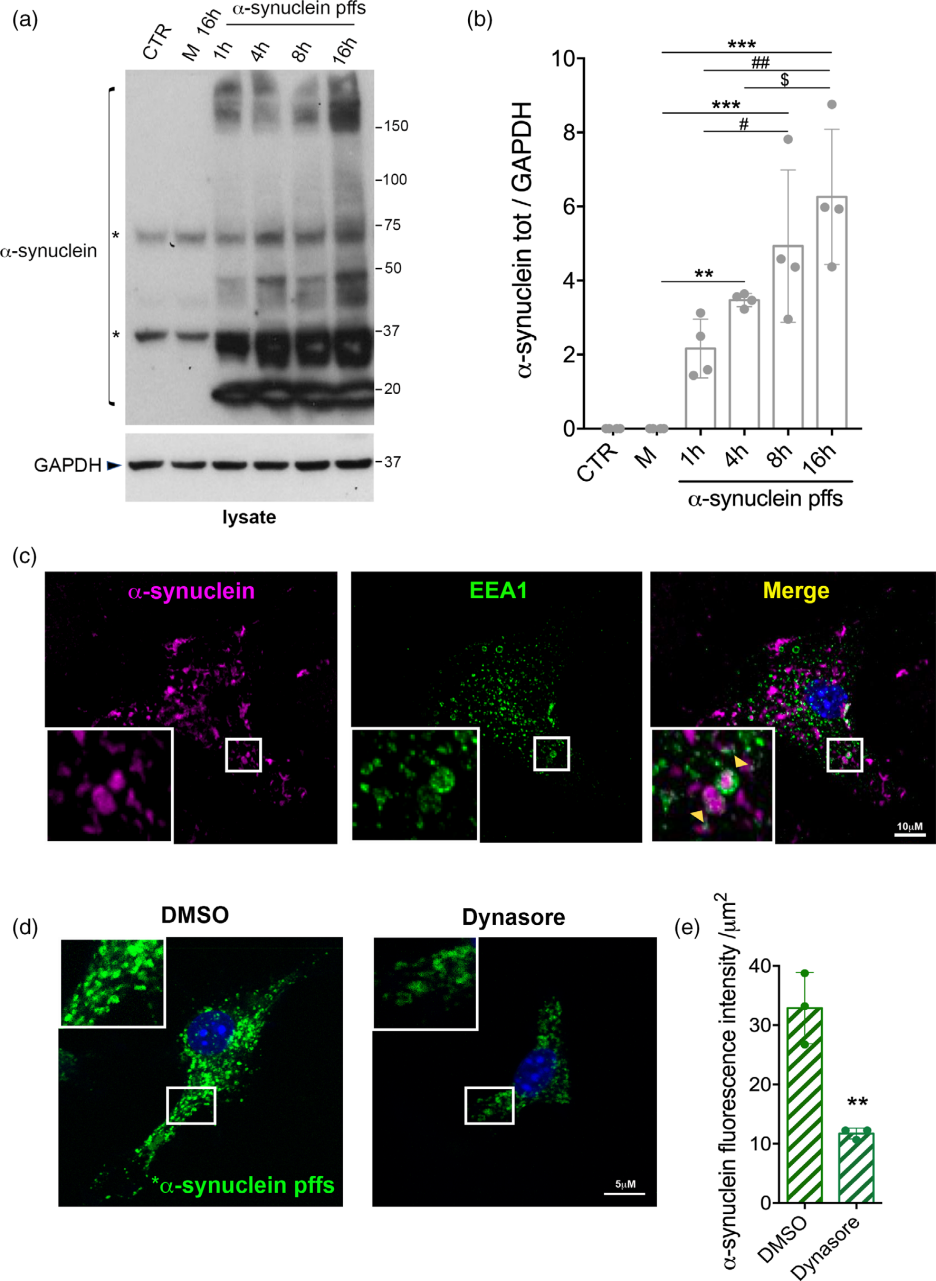
Primary astrocytes take up α‐synuclein preformed fibrils (pffs) through dynamin‐dependent endocytosis. (a) Cell lysates of primary astrocytes treated with α‐synuclein pffs for different times (1, 4, 8, and 16 hr) were subjected to immunoblotting using α‐synuclein and GAPDH antibodies. Monomeric α‐synuclein (M) for 16 hr and untreated cells were used as control. Asterisks in the α‐synuclein immunoblot indicate α‐synuclein nonspecific bands. (b) Quantification of α‐synuclein is normalized to GAPDH protein. Data are representative of four independent experiments and are expressed as the mean ± *SD*. Data were analyzed using one‐way analysis of variance (ANOVA) followed by Tukey's post hoc test. **p = .0061, α‐synuclein pffs 4 hr versus α‐synuclein M; ***p = .0002, α‐synuclein pffs 8 hr versus α‐synuclein M; ***p < .0001, α‐synuclein pffs 16 hr versus α‐synuclein M; ^#^ *p* = .036, α‐synuclein pffs 8 hr versus α‐synuclein pffs 1 hr; ^##^ *p* = .0012, α‐synuclein pffs 16 hr versus α‐synuclein pffs 1 hr; ^$^ *p* = .0341, α‐synuclein pffs 16 hr versus α‐synuclein pffs 4 hr. (c) Maximum intensity Z‐projection confocal images of primary astrocytes treated with α‐synuclein pffs for 16 hr and stained for α‐synuclein (purple), Early Endosome Antigen 1 (EEA1) (green) and nuclei with DAPI (blue). Scale bar 10 μm. (d) Maximum intensity Z‐projection confocal images of primary astrocytes treated with *α‐synuclein pffs and Dynasore, or DMSO as control, for 4 hr. Scale bar 5 μm. (e) Quantification of *α‐synuclein pffs is shown as mean of fluorescence intensity from three independent experiments (~50 cells per experiment). Quantification of *α‐synuclein pffs is calculated as fluorescence intensity divided by the cell area (μm^2^). Data are expressed as the mean ± *SD* and analyzed by unpaired *t* test; **p = .0038. Individual points of the graphs represent each single experiment [Color figure can be viewed at wileyonlinelibrary.com]

Astrocytes are known to be actively involved in the clearance of various disease‐specific protein aggregates (Jung & Chung, 2018). To understand whether astrocytes were able to clear PD‐related aggregates, we first performed a time‐course experiment where primary astrocytes were treated for 1, 4, 8, and 16 hr with pffs. Monomeric α‐synuclein and untreated cells were used as control. Quantifying total α‐synuclein internalized by the cells, we confirmed that astrocytes take up α‐synuclein pffs and that the amount of internalized α‐synuclein significantly increases with the time of treatment (Figure 1a,b).

Next, we examined endocytosis as a candidate mechanism for the uptake of pffs. We observed that following treatment with pffs for 16 hr, primary astrocytes displayed intracellular α‐synuclein that partially colocalized with EEA1‐positive early endosomes (Figure 1c). To further dissect the mechanism underlying α‐synuclein endocytosis, we used Dynasore, a chemical blocker of dynamin‐dependent endocytosis (Macia et al., 2006), and labeled α‐synuclein pffs with an amine‐reactive pH‐sensitive dye (*α‐synuclein pffs) that increases in fluorescence as the pH of their surroundings become more acidic, including late endosomes and lysosomes. We noted that the fluorescence intensity of intracellular *α‐synuclein pffs was lower after Dynasore treatment (Figure 1d,e). Collectively, these results demonstrate that the internalization of pffs occurs via dynamin‐dependent endocytosis in astrocytes.

### 3.2 Extracellular clusterin interacts with α‐synuclein pffs

**FIGURE 2:**
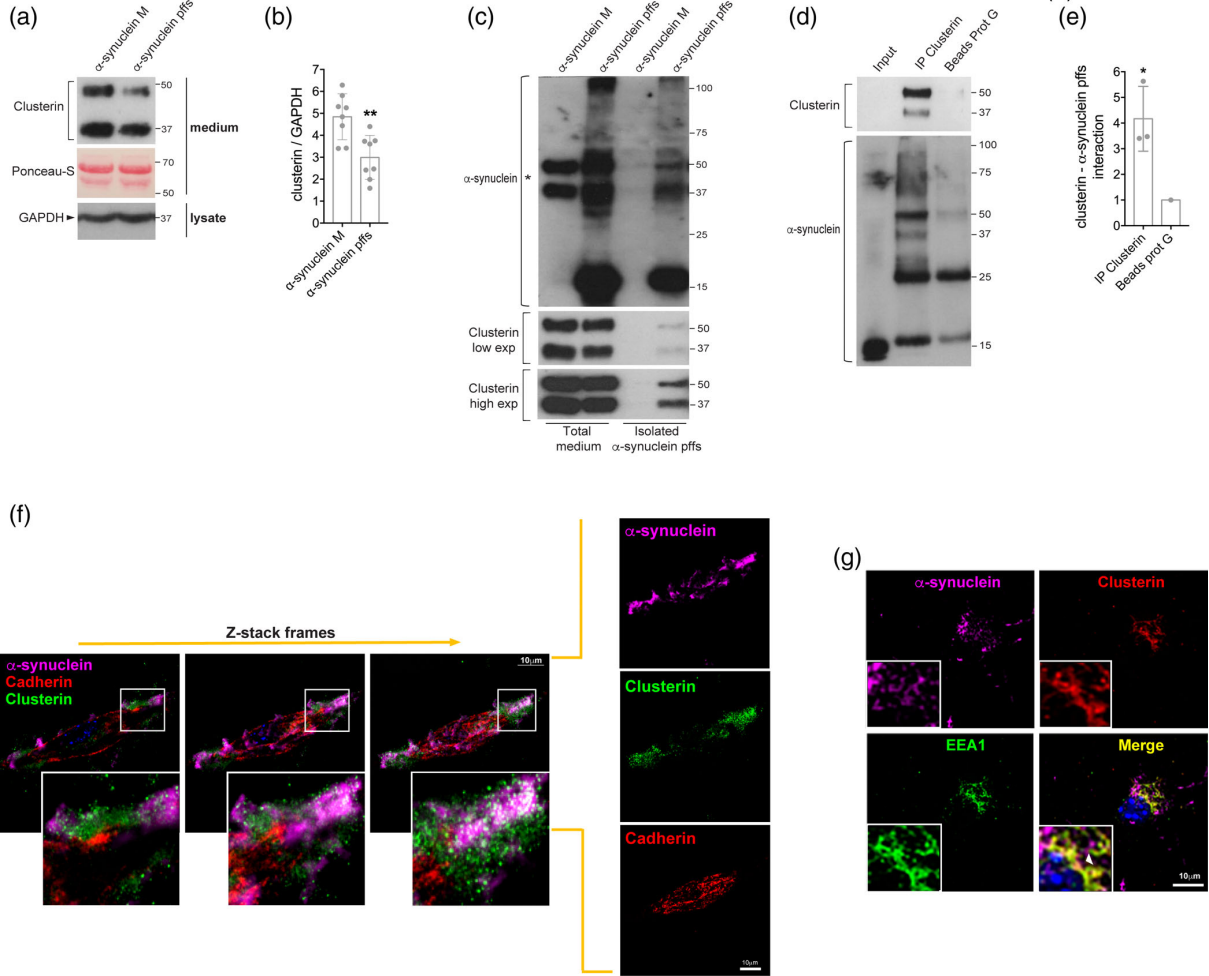
Extracellular clusterin interacts with α‐synuclein preformed fibrils (pffs). (a) Medium from primary astrocytes treated with α‐synuclein pffs for 16 hr, or with monomeric α‐synuclein (m), were subjected to immunoblotting using clusterin antibody. Ponceau‐S staining was used as a loading control. (b) Quantification of clusterin is normalized to GAPDH protein of cell lysates. Data are from eight independent experiments and are expressed as the mean ± *SD*. Data were analyzed using unpaired *t* test; **p = .0028. (c) α‐Synuclein pffs isolated by centrifugation from total medium of primary astrocytes treated with α‐synuclein pffs, or monomeric α‐synuclein (M), for 16 hr were subjected to immunoblotting using clusterin and α‐synuclein antibodies. Asterisk in the α‐synuclein immunoblot indicates clusterin bands still present after Clusterin antibody stripping. (d) Clusterin immunoprecipitated from the medium of primary astrocytes treated with α‐synuclein pffs for 16 hr was subjected to immunoblotting using an α‐synuclein antibody. (e) Quantification of clusterin—α‐synuclein pffs interaction has been obtained by normalization of α‐synuclein pffs bound to clusterin for α‐synuclein pffs bound to protein‐G beads. Data are representative of three independent experiments and are expressed as the mean ± *SD*. Data were analyzed using one‐sample *t* test; *p = .0493. (f) Z‐stack frames of primary astrocytes treated with α‐synuclein pffs for 16 hr and stained for α‐synuclein (purple), clusterin (green), cadherin (red), and nuclei with DAPI (blue). Scale bar 10 μm. (g) Maximum intensity Z‐projection confocal images of primary astrocytes treated with α‐synuclein pffs for 16 hr and stained for α‐synuclein (purple), clusterin (red), Early Endosome Antigen 1 (EEA1) (green) and nuclei with DAPI (blue). Scale bar 10 μm. Individual points of the graphs represent each single experiment [Color figure can be viewed at wileyonlinelibrary.com]

The extracellular chaperone clusterin has been reported to interact with aggregated amyloid proteins, including α‐synuclein oligomeric species in cell‐free systems (Whiten et al., 2018; Yerbury et al., 2007), and to interfere with the uptake and clearance of extracellular beta‐amyloid aggregates by astrocytes (Mulder et al., 2014; Nielsen et al., 2010). We therefore explored whether clusterin was involved in the uptake of α‐synuclein pffs by astrocytes. We first analyzed the level of extracellular clusterin in the media of primary astrocytes exposed to pffs or monomeric α‐synuclein as a control. Extracellular clusterin levels were lower in the culture media of cells treated with α‐synuclein pffs compared to cells treated with monomeric protein (Figure 2a,b), suggesting that clusterin may have interacted and been internalized with α‐synuclein pffs. We did not find any modulation of extracellular clusterin in the media of astrocytes exposed to monomeric α‐synuclein when compared with untreated cells (Supplementary Figure S1), indicating that clusterin is specifically modulated by aggregated α‐synuclein.

Based on these observations, we next asked whether extracellular clusterin was able to bind α‐synuclein pffs. We treated primary astrocytes with pffs, or monomeric α‐synuclein as control, and 16 hr after the treatment we isolated insoluble α‐synuclein pffs from the total media by centrifugation. Clusterin cosedimented with α‐synuclein pffs (Figure 2c), suggesting that clusterin binds pffs in the extracellular compartment. We then confirmed a physical interaction between the proteins by immunoprecipitation of clusterin from the media of treated astrocytes and by colocalization of clusterin and α‐synuclein through immunostaining. We found that clusterin interacts with α‐synuclein pffs in the media of treated astrocytes (Figure 2d,e) and colocalizes in proximity to the plasma membrane (Figure 2f). Taken together, these multiple orthogonal techniques demonstrate that clusterin binds α‐synuclein pffs in the extracellular compartment.

Since our findings indicate that clusterin could undergo endocytosis with pffs, we explored whether a complex of clusterin and pffs could be detectable within cells. We found clusterin and pffs partially colocalized with EEA1‐positive endosomes, indicating that complexes can be internalized by astrocytes through the endocytic pathway (Figure 2g). In addition, we noted that clusterin mostly appears with a cytoplasmatic localization as it is synthesized as a precursor and then cleaved in two chains (α and β) prior secretion (Foster et al., 2019).

### 3.3 Extracellular clusterin limits the uptake of α‐synuclein pffs by primary murine astrocytes

**FIGURE 3:**
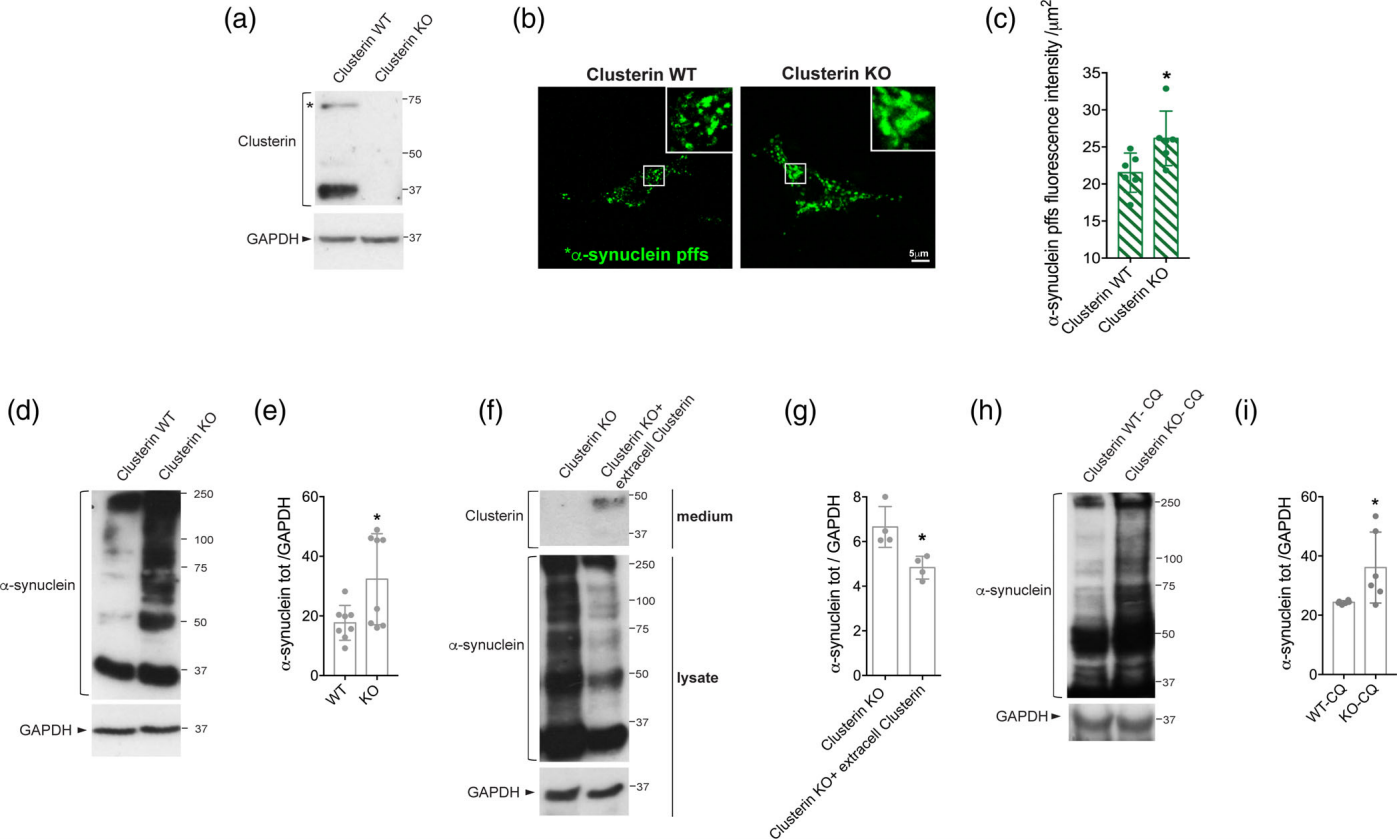
Extracellular clusterin limits the uptake of α‐synuclein preformed fibrils (pffs) by murine primary astrocytes. (a) Cell lysates from clusterin wild‐type (WT) and knock‐out (KO) astrocytes were subjected to immunoblotting using clusterin and GAPDH antibodies. Asterisk indicates clusterin precursor. (b) Maximum intensity Z‐projection confocal images of clusterin WT and KO astrocytes treated with *α‐synuclein pffs for 4 hr. Scale bar 5 μm. (c) Quantification of *α‐synuclein pffs is shown as mean of fluorescence intensity from six independent experiments (~50 cells per experiment). Quantification of *α‐synuclein pffs is calculated as fluorescence intensity divided by the cell area (μm^2^). Data are expressed as the mean ± *SD*. Data were analyzed using unpaired *t* test; *p = .0315. (d) Cell lysates from clusterin WT and KO primary astrocytes treated with pffs for 16 hr were subjected to immunoblotting using α‐synuclein and GAPDH antibodies. (e) Quantification of intracellular α‐synuclein is normalized to GAPDH protein. Data are representative of eight independent experiments and are expressed as the mean ± *SD*. Data were analyzed using unpaired *t* test; *p = .0239. (f) Cell lysates from clusterin KO primary astrocytes treated with pffs for 16 hr in a medium containing or not containing extracellular clusterin were subjected to immunoblotting using α‐synuclein, clusterin and GAPDH antibodies. (g) Quantification of intracellular α‐synuclein is normalized to GAPDH protein. Data are representative of four independent experiments and are expressed as the mean ± *SD*. Data were analyzed using unpaired *t* test; *p = .0134. (h) Cell lysates from clusterin WT and KO primary astrocytes treated with pffs and chloroquine (CQ) for 16 hr were subjected to immunoblotting using α‐synuclein and GAPDH antibodies. (i) Quantification of intracellular α‐synuclein normalized to GAPDH protein. Data are representative of six independent experiments and are expressed as the mean ± *SD*. Data were analyzed using unpaired *t* test; *p = .0377. Individual points of the graphs represent each single experiment [Color figure can be viewed at wileyonlinelibrary.com]

It has been shown that clusterin can positively or negatively affect beta‐amyloid internalization depending on the conformational state of the protein and the cell type investigated (Hammad et al., 1997; Mulder et al., 2014; Nielsen et al., 2010; Zlokovic et al., 1996). In astrocytes, extracellular clusterin can decrease the uptake of beta‐amyloid oligomers and fibrils (Mulder et al., 2014; Nielsen et al., 2010). Based on these observations, we explored the effect of clusterin on the endocytosis of α‐synuclein pffs using primary astrocytes from clusterin WT or KO mice (Figure 3a). As shown in Figure 3b,c, clusterin KO astrocytes had higher amounts of intracellular *α‐synuclein pffs compared to WT cells. We confirmed the increased uptake of α‐synuclein pffs observed in clusterin KO astrocytes using immunoblotting analysis (Figure 3d,e). To confirm that extracellular clusterin is sufficient for these effects, we treated clusterin KO astrocytes with a medium containing (conditioned medium from WT cells) or not containing (conditioned medium of KO cells) extracellular clusterin in the presence of α‐synuclein pffs. Importantly, supplementing clusterin in the medium was able to reverse the increased pffs uptake by clusterin KO astrocytes (Figure 3f,g).

To further establish that extracellular clusterin affects endocytosis, we quantified the total intracellular α‐synuclein in clusterin WT and KO astrocytes after blocking lysosomal degradation with CQ. There was a significant increase in α‐synuclein uptake by clusterin KO astrocytes compared to WT cells after treatment with CQ (Figure 3h,i), confirming that clusterin acts at level of the endocytosis. Overall, these results show that extracellular clusterin, through binding to pffs, limits the uptake of α‐synuclein by astrocytes.

### 3.4 Conservation of clusterin‐dependent α‐synuclein pffs uptake from mouse to human astrocytes

We next explored whether clusterin was able to influence the uptake of pffs in human cells using iPSC‐derived astrocytes (hiAstrocytes). These hiAstrocytes expressed key astrocyte markers, including S100β, GFAP, and EAAT2, and not neuronal markers including TubβIII antibody (Supplementary Figure S3a,b). Additionally, we determined the functional properties of these cells by examining their capacity to propagate intracellular Ca^2+^ waves, which are important for neuron–glia and glia–glia communication (Scemes & Giaume, 2006), and to support neurite growth (di Domenico et al., 2019; Li et al., 2018). Using the Fluo4‐AM, we observed that hiAstrocytes display intracellular Ca^2+^ fluctuation under basal conditions (Supplementary Figure S3c). Moreover, by quantification of dendrite branching, we found that murine dopaminergic neurons cocultured with hiAstrocytes exhibit longer and a higher number of dendrites compared to neurons cultured alone (Supplementary Figure S3d,e). Overall, these results indicate that our hiAstrocytes are functional.

**FIGURE 4:**
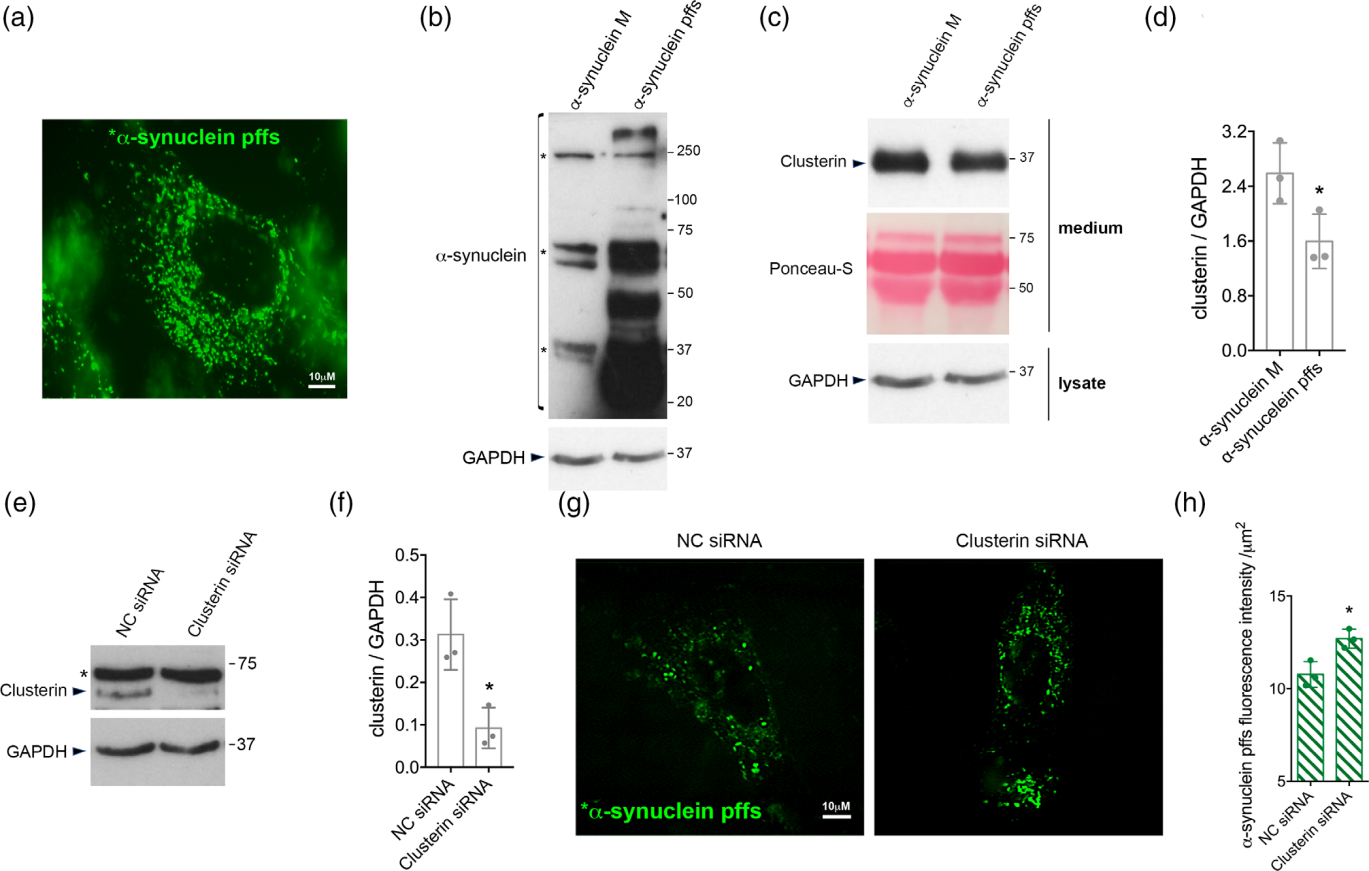
Conservation of clusterin‐dependent α‐synuclein preformed fibrils (pffs) uptake from mouse to human astrocytes. (a) Representative image of hiAstrocytes (27 days of differentiation) treated with *α‐synuclein pffs for 48 hr. Scale bar 10 μm. (b) Cell lysates from hiAstrocytes (27 days of differentiation) treated with α‐synuclein pffs, or monomeric α‐synuclein (m), for 48 hr were subjected to immunoblotting using α‐synuclein and GAPDH antibodies. Asterisks in the α‐synuclein immunoblot indicate α‐synuclein nonspecific bands. (c) Medium from hiAstrocytes (27 days of differentiation) treated with α‐synuclein pffs, or monomeric α‐synuclein (M), for 48 hr were subjected to immunoblotting using clusterin antibody. Ponceau‐S staining was used as loading control. (d) Quantification of clusterin is normalized to GAPDH protein of cell lysates. Data are representative of three independent experiments and are expressed as the mean ± *SD*. Data were analyzed using unpaired *t* test; *p = .0446. (e) Cell lysates from hiAstrocytes (31 days of differentiation) transfected with negative control (NC) or clusterin siRNA for 24 hr were subjected to immunoblotting using clusterin and GAPDH antibodies. Asterisk in the clusterin immunoblot indicates clusterin nonspecific band. (f) Quantification of clusterin precursor is normalized to GAPDH. Data are representative of three independent experiments and are expressed as the mean ± *SD*. Data were analyzed using unpaired *t* test; *p = .0165. (g) Representative images of hiAstrocytes (35 days of differentiation) transfected with NC or clusterin siRNA and treated with *α‐synuclein pffs for 24 hr. Scale bar 10 μm. (h) Quantification of *α‐synuclein pffs is shown as mean of fluorescence intensity from three independent experiments (~50 cells per experiment). Quantification of *α‐synuclein pffs is calculated as fluorescence intensity divided by the cell area (μm^2^). Data are expressed as the mean ± *SD*. Data were analyzed using unpaired *t* test; *p = .0178. Individual points of the graphs represent each single experiment [Color figure can be viewed at wileyonlinelibrary.com]

Subsequently, we investigated whether hiAstrocytes were able to internalize α‐synuclein pffs. We treated hiAstrocytes with unlabeled or *α‐synuclein pffs for 48 hr and we were able to readily detect intracellular pffs (Figure 4a,b). Next, we explored whether extracellular clusterin was modulated upon α‐synuclein pffs exposure. As in primary murine astrocytes, we found a significant decrease in the amount of clusterin in the media after exposure to pffs compared to monomeric protein (Figure 4c,d). Finally, we examined whether clusterin was able to limit α‐synuclein pffs uptake by human astrocytic cells. We lowered clusterin expression in hiAstrocytes using siRNA (Figure 4e,f) and quantified intracellular *α‐synuclein pffs. Importantly, we found increased *α‐synuclein pffs internalized in clusterin KD hiAstrocytes compared to control cells (Figure 4g,h). Collectively, these findings indicate that clusterin limits the uptake/clearance of α‐synuclein pffs by human astrocytes as in murine cells.

### 3.5 Clusterin protein levels are modulated in two animal models relevant to PD

**FIGURE 5:**
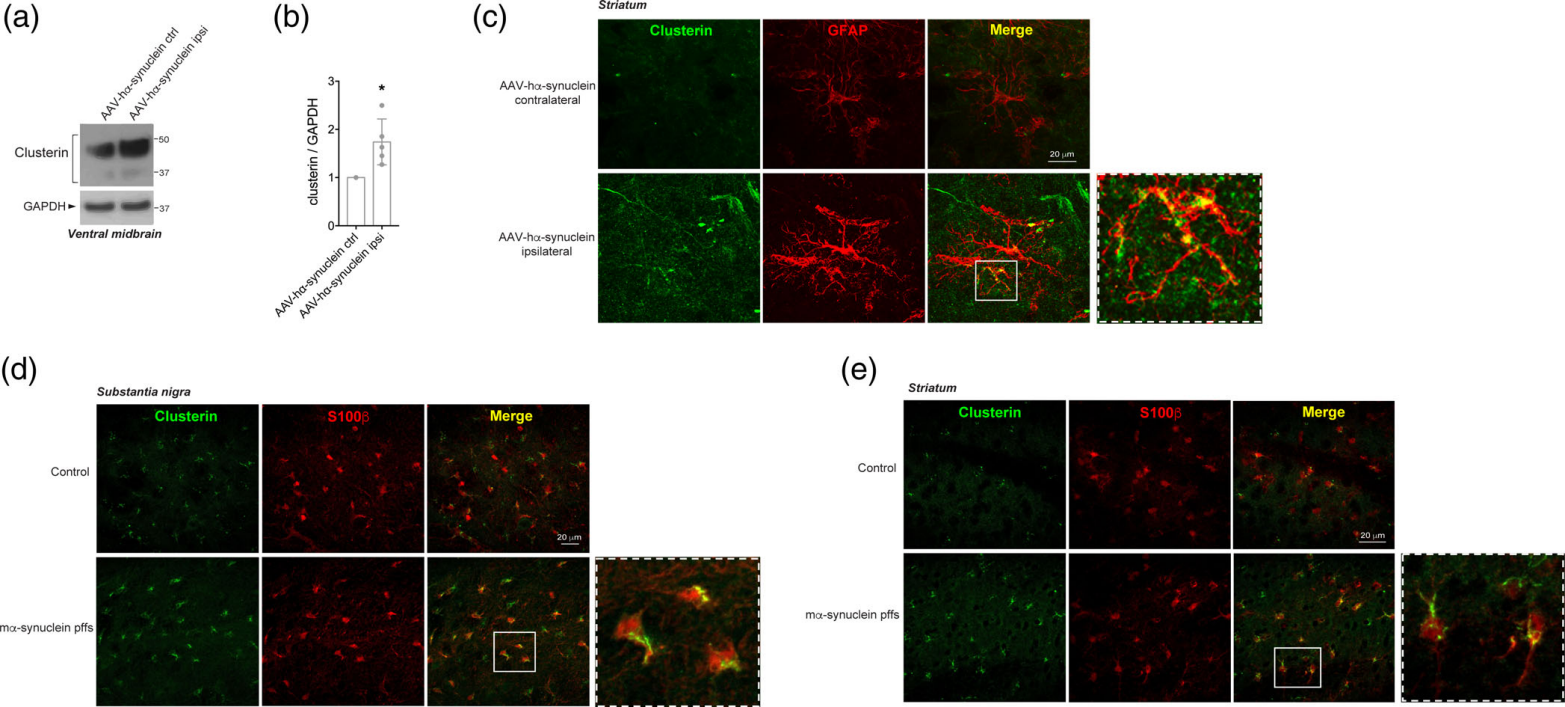
Clusterin protein levels are increased in two different animal models of Parkinson's disease. (a) Lysates from contralateral and ipsilateral ventral midbrain of mice injected with adeno‐associated virus (AAV)‐hα‐synuclein and sacrificed after 8 weeks were subjected to immunoblotting using clusterin and GAPDH antibodies. (b) Quantification of total cleaved clusterin is normalized to GAPDH protein. Data are representative of five animals and are expressed as the mean ± *SD*. Data were analyzed using one‐sample *t* test; *p = .0253. (c) Representative images of clusterin‐GFAP double labeling in the ipsilateral and contralateral striatum of mice injected with AAV‐hα‐synuclein and sacrificed after 8 weeks. Scale bar 20 μm. (d) Representative images of clusterin‐S100β double labeling in the substantia nigra of mice injected with α‐synuclein preformed fibrils (pffs) and sacrificed after 4 weeks. Scale bar 20 μm. (e) Representative images of clusterin‐S100β double labeling in the striatum of mice injected with α‐synuclein pffs and sacrificed after 4 weeks. Scale bar 20 μm [Color figure can be viewed at wileyonlinelibrary.com]

To determine if clusterin is also affected by α‐synuclein in vivo, we analyzed clusterin expression in two PD animal models. Injection of either AAV‐hα‐synuclein and α‐synuclein pffs in vivo reproduces many of the characteristic features of PD, including abnormal α‐synuclein aggregation, dopaminergic neuronal degeneration and motor behavior impairment (Faustini et al., 2018; Luk et al., 2012; Ulusoy, Decressac, Kirik, & Björklund, 2010). By both western blotting and immunofluorescence analysis, we detected an increased amount of total clusterin in the ventral midbrain and striatum of mice injected with AAV‐hα‐synuclein (Figure 5a–c) as well as in mice injected with α‐synuclein pffs (Figure 5d,e). Moreover, in both animal models we found clusterin expressed in astrocytes (Figure 5c–e), indicating that α‐synuclein‐mediated modulation of clusterin may occur in astrocytes in vivo. These congruent results in two different models argue that the increment of clusterin protein levels may have a pivotal role in the pathology of PD.

## 4 DISCUSSION

Accumulating literature indicates that aggregated α‐synuclein species released by degenerating neurons can actively contribute to the spreading of PD pathology (Bieri et al., 2018; Lopes da Fonseca et al., 2015). Therefore, the clearance of extracellular α‐synuclein toxic forms is crucial to control the propagation and the progression of the disease. Here, we provide evidence that the extracellular chaperone clusterin binds to and limits the clearance of α‐synuclein pffs by murine and human astrocytes.

It is known that microglia and astrocytes can phagocytose and clear disease‐specific protein aggregates, including α‐synuclein, in various experimental systems (Filippini et al., 2019; Jung & Chung, 2018). Supporting potential relevance to human disease, two independent studies reported the presence of astrocytic α‐synuclein‐immunoreactive inclusions in postmortem brains of PD patients (Braak et al., 2007; Wakabayashi et al., 2000). Moreover, Braak et al. (2007) observed that the density and location of α‐synuclein‐containing inclusions appear to correlate with the presence of neuronal inclusion bodies. Such observations suggest that a major pathway for the removal of α‐synuclein species released by degenerating neurons may be via astrocytes, thus limiting the spread of pathogenesis. Here, we have explored whether astrocytes were able to internalize α‐synuclein pffs and outlined the molecular mechanism behind this process. We show that primary murine and hiPSC‐derived astrocytes can take up α‐synuclein pffs and that the internalization occurs through dynamin‐dependent endocytosis. These results indicate that astrocytes are actively involved in the clearance of extracellular PD‐related fibrils and suggest that their dysfunctions may contribute to the propagation of α‐synuclein toxic forms and, hence, to PD pathology.

Molecular chaperones may control the accumulation and the clearance of misfolded/amyloid proteins in the extracellular space. Although several extracellular chaperones have been reported to affect the uptake of Alzheimer's disease‐related aggregates (Hammad et al., 1997; Mulder et al., 2014; Nielsen et al., 2010; Zlokovic et al., 1996), little is known about their effects on α‐synuclein. Our findings demonstrate that extracellular clusterin reduces the uptake of α‐synuclein fibrils by astrocytes. Specifically, we found lower levels of extracellular clusterin in the culture media of cells treated with α‐synuclein pffs compared to cells treated with monomeric protein, suggesting a binding of clusterin to pffs that are typically endocytosed by the cells. It is known that molecular chaperones can either modulate the aggregation state or affect the clearance of misfolded/aggregated proteins through physical interaction (Wilson, Yerbury, & Poon, 2008; Yerbury et al., 2005). Therefore, we tested the hypothesis that the decrease in extracellular clusterin levels observed in response to pffs was due to its interaction with the endocytosed fibrils. Using several orthogonal approaches, we demonstrated that clusterin interacts with α‐synuclein pffs in the extracellular compartment and that clusterin/pffs complexes can be internalized by astrocytes through the endocytic pathway. Our results indicate that clusterin is able to interact with α‐synuclein fibrils likely by binding their exposed hydrophobic regions as previously reported for α‐synuclein oligomeric species (Whiten et al., 2018) and for the Hsp27 chaperone complexed with α‐synuclein fibrils (Cox et al., 2018).

**FIGURE 6:**
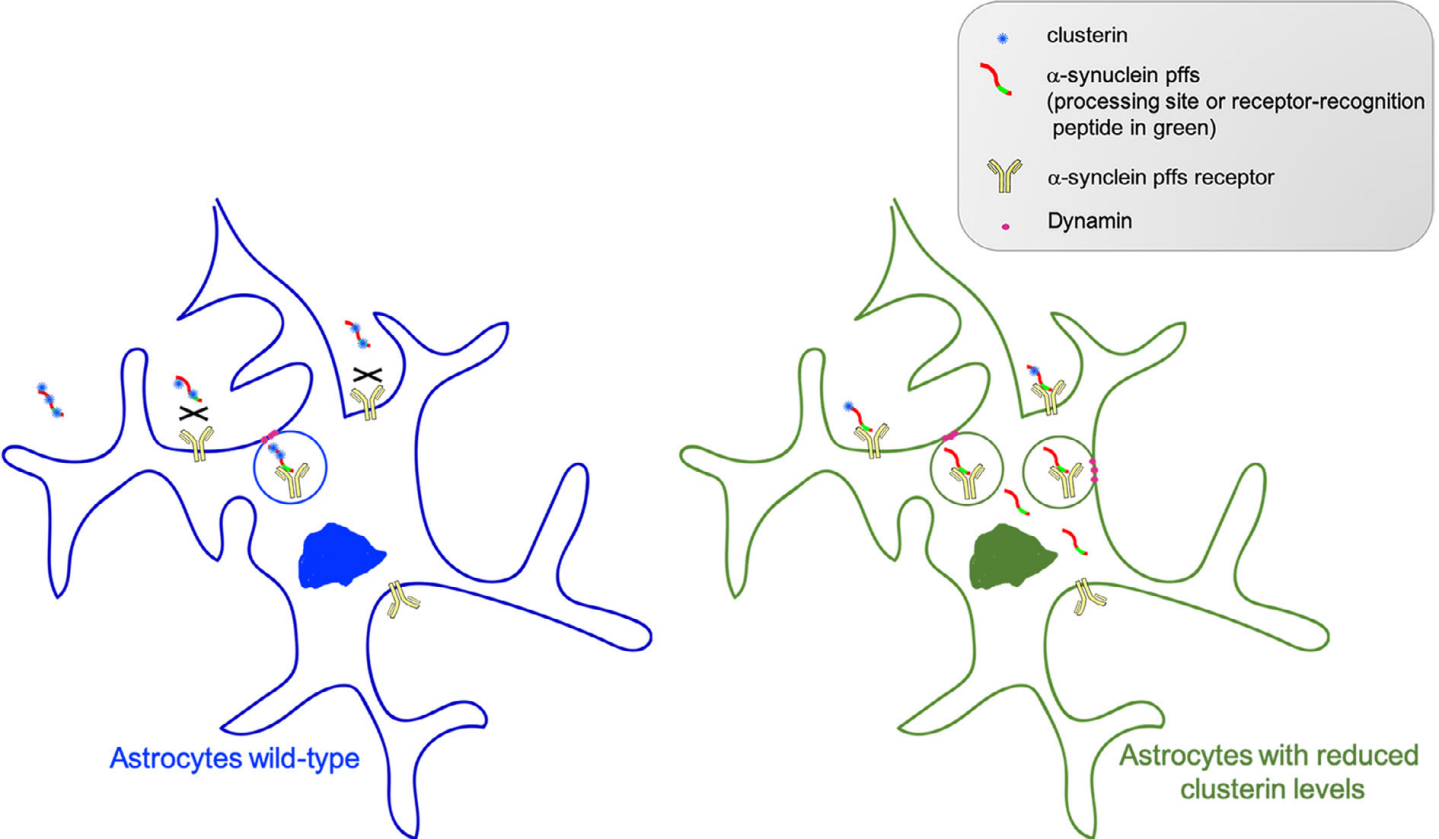
Schematic hypothesis. Extracellular clusterin binds to α‐synuclein preformed fibrils (pffs) and masks the pffs processing site, associated with the endocytosis, or the receptor‐recognition peptide, thus limiting pffs uptake by astrocytes. Diminished expression of clusterin levels allows α‐synuclein pffs to be processed and/or recognized by the surface receptor(s), thus improving the clearance of α‐synuclein pffs by astrocytes [Color figure can be viewed at wileyonlinelibrary.com]

To gain more insights into the effect of clusterin on the uptake/clearance of α‐synuclein pffs, we took advantage of clusterin KO astrocytes. Of interest, we observed an increased amount of internalized pffs in KO cells compared to their WT counterparts. Furthermore, we found that the supplement of extracellular clusterin was able to rescue the effects of clusterin deficiency in astrocytes. These findings demonstrate that clusterin limits the endocytosis of α‐synuclein pffs by astrocytes. In line with these results, it has been reported that the preincubation of clusterin with beta‐amyloid oligomers or fibrils reduces the amount of beta‐amyloid aggregates internalized by human astrocytes (Mulder et al., 2014; Nielsen et al., 2010), supporting that clusterin interferes with the uptake and clearance of α‐synuclein fibrils. Additional studies will allow to understand whether clusterin could limit the uptake of other α‐synuclein species, including oligomers. One model consistent with our observations is that, by binding to α‐synuclein pffs, clusterin might mask the α‐synuclein pffs receptor‐recognition site or a processing site associated with the endocytosis, thus impeding their uptake and clearance (Figure 6). This, in turn, could lead to an higher concentration of aggregated α‐synuclein in the extracellular space, which might induce the spreading of α‐synuclein toxic species between neurons. In this regard, it would be important to understand if genetic deletion or down‐regulation of clusterin levels in vivo would improve the clearance of aggregated α‐synuclein by astrocytes, protect neurons from the spreading of toxic forms of α‐synuclein and thus be neuroprotective for PD. To date, it is controversial if clusterin is beneficial (Boggs et al., 1996; Gregory et al., 2017; Qi, Wang, Chu, Li, & Ma, 2018; Whiten et al., 2018) or detrimental (DeMattos et al., 2002; DeMattos et al., 2004; Mulder et al., 2014; Nielsen et al., 2010) during pathological conditions.

Another outstanding question is whether clusterin could interfere with the uptake of α‐synuclein pffs even in other brain immune cells, like microglia, or if this occurs specifically in astrocytes to likely preserve their physiological roles. In this context, an enhanced clearance of α‐synuclein might lead astrocytes toward a reactive phenotype with impaired physiological functions, which, in turn, could further damage neurons and contribute to the pathology (Lee, Kim, & Lee, 2010; Lee, Suk, et al., 2010). Future research aimed at understanding the underlying mechanism and/or receptor(s) involved in α‐synuclein pffs uptake will fully define the process by which glial cells can clear this protein. Several receptors have been described to be involved in the endocytosis of α‐synuclein species and available evidence indicates that the uptake of α‐synuclein might be conformation‐ and cell type‐dependent (Filippini et al., 2019). In this regard, little is known about the receptors specifically involved in the α‐synuclein aggregates uptake by astrocytes. It has been observed that, in contrast to what occurs in neurons or in microglial cells, in astrocytes α‐synuclein aggregates endocytosis is not mediated through toll‐like receptor 4 (Fellner et al., 2013; Rannikko, Weber, & Kahle, 2015), heparan sulfate (Ihse et al., 2017) or lymphocyte‐activation gene 3 (Mao et al., 2016).

Finally, using the AAV‐hα‐synuclein overexpression and pffs injections in vivo, we confirmed that clusterin levels are increased in the substantia nigra and in the striatum of both PD animal models. Overall, our findings indicate that clusterin is modulated even in vivo and that the increment of its levels may be crucial for the spreading of α‐synuclein species throughout the brain. However, it will be important to understand if the modulation of clusterin in the AAV‐based model is related to the α‐synuclein spreading, given that the expression and accumulation of α‐synuclein occurs directly in neurons in this model. In contrast to what observed in the α‐synuclein pffs‐injected model, it still unclear if α‐synuclein aggregates spread between neurons in the AAV model (Volpicelli‐Daley, Kirik, Stoyka, Standaert, & Harms, 2016).

Although further investigations are required to better dissect the role of clusterin in PD pathogenesis, our findings reveal that the extracellular chaperone clusterin binds to and limits the uptake and clearance of α‐synuclein pffs by murine and human astrocytes. Of particular relevance, our observations suggest that clusterin might actively contribute to the spreading of α‐synuclein pathology in PD.

## CONFLICT OF INTEREST

The authors declare no conflict of interest.

## Supporting information


**Appendix S1**: Supporting information

## Data Availability

The data that support the findings of this study are available from the corresponding author upon reasonable request.
