# Supplementary material for: Extracellular clusterin limits the uptake of α‐synuclein fibrils by murine and human astrocytes
Source: Glia. 2020 Oct 12;69(3):681–96. doi: 10.1002/glia.23920 (PMC7821254; doi:10.1002/glia.23920)
Supplement: Supplementary file 1 — Appendix S1: Supporting information [file GLIA-69-681-s001.docx]

**Supplementary information for**

**Extracellular clusterin limits the uptake of α-synuclein fibrils by murine and human astrocytes**

**Running title:** Clusterin limits α-synuclein uptake

Alice Filippini^1,*^, Veronica Mutti^2^, Gaia Faustini^2^, Francesca Longhena^2^, Ileana Ramazzina^3,^ Federica Rizzi^3^, Alice Kaganovich^4^, Dorien A Roosen^4^, Natalie Landeck^4^, Megan Duffy^4^, Isabella Tessari^5^, Federica Bono^6^, Chiara Fiorentini^2^, Elisa Greggio^5^, Luigi Bubacco^5^, Arianna Bellucci^6^, Mariacristina Missale^2^**,** Mark R Cookson^4^, Massimo Gennarelli^1,7^, Isabella Russo^1,7,CA^

^CA^ To whom correspondence should be addressed:

Isabella Russo PhD, Unit of Biology and Genetics, Department of Molecular and Translational Medicine, University of Brescia, Viale Europa 11, 25123, Brescia, Italy.

Tel. +390303717255; Fax +390303701157; e-mail: isabella.russo@unibs.it.


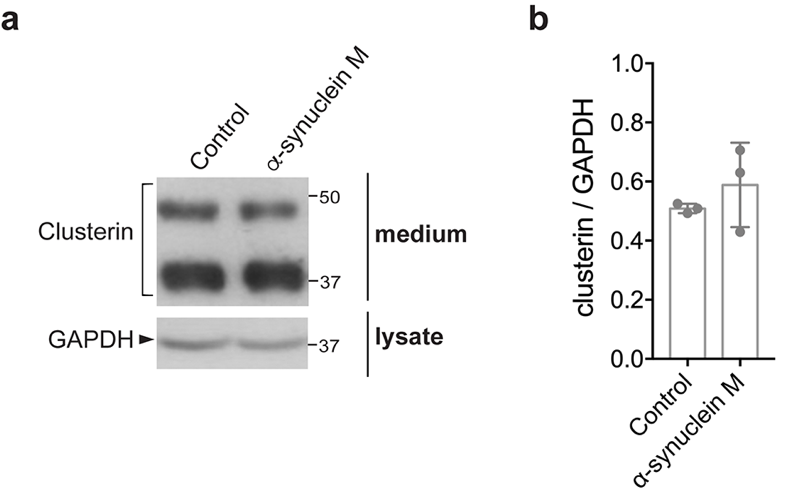


**Supplementary Figure 1: Extracellular clusterin is not modulated by monomeric α-synuclein. (a)** Medium from primary astrocytes treated with monomeric (M) α-synuclein for 16h, or left untreated, were subjected to immunoblotting using clusterin antibody. **(b)** Quantification of clusterin is normalized to GAPDH protein of cell lysates. Data are from three independent experiments and are expressed as the mean ± SD; individual points represent each single experiment.


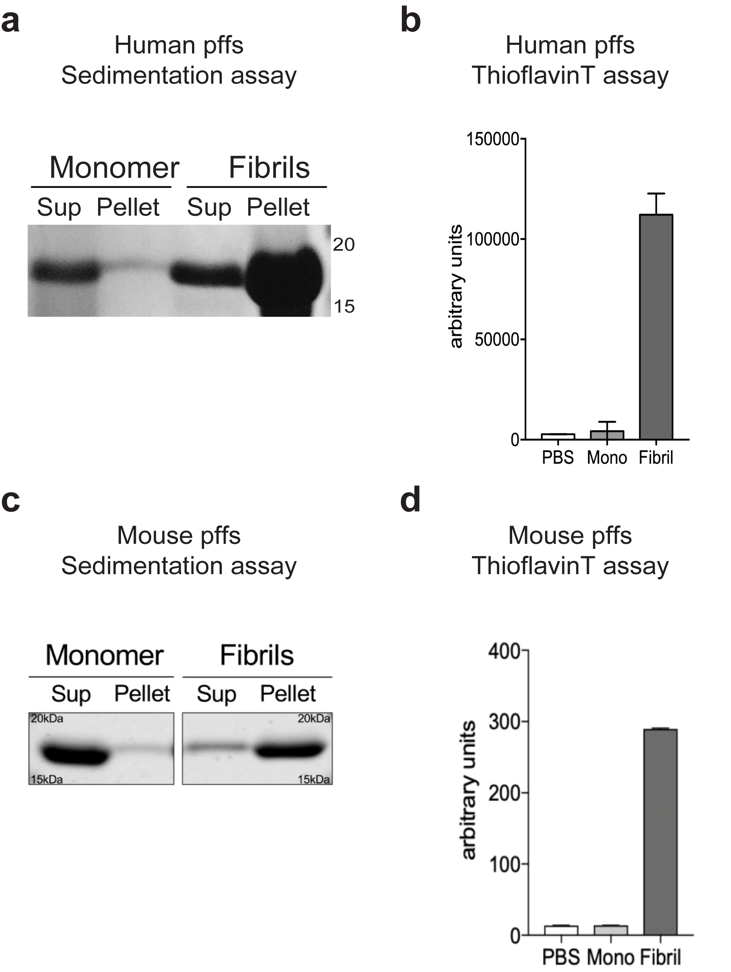


**Supplementary Figure 2. Quality control assays to verify α-synuclein pffs formation. (a, c)** Sedimentation assay performed on human and mouse α-synuclein pffs confirms greater amounts of protein in the pellet vs. the supernatant fraction of fibrils. **(b, d)** ThT assay performed on human and mouse α-synuclein pffs shows high levels of ThT fluorescence signal with pffs compared to monomer and PBS.


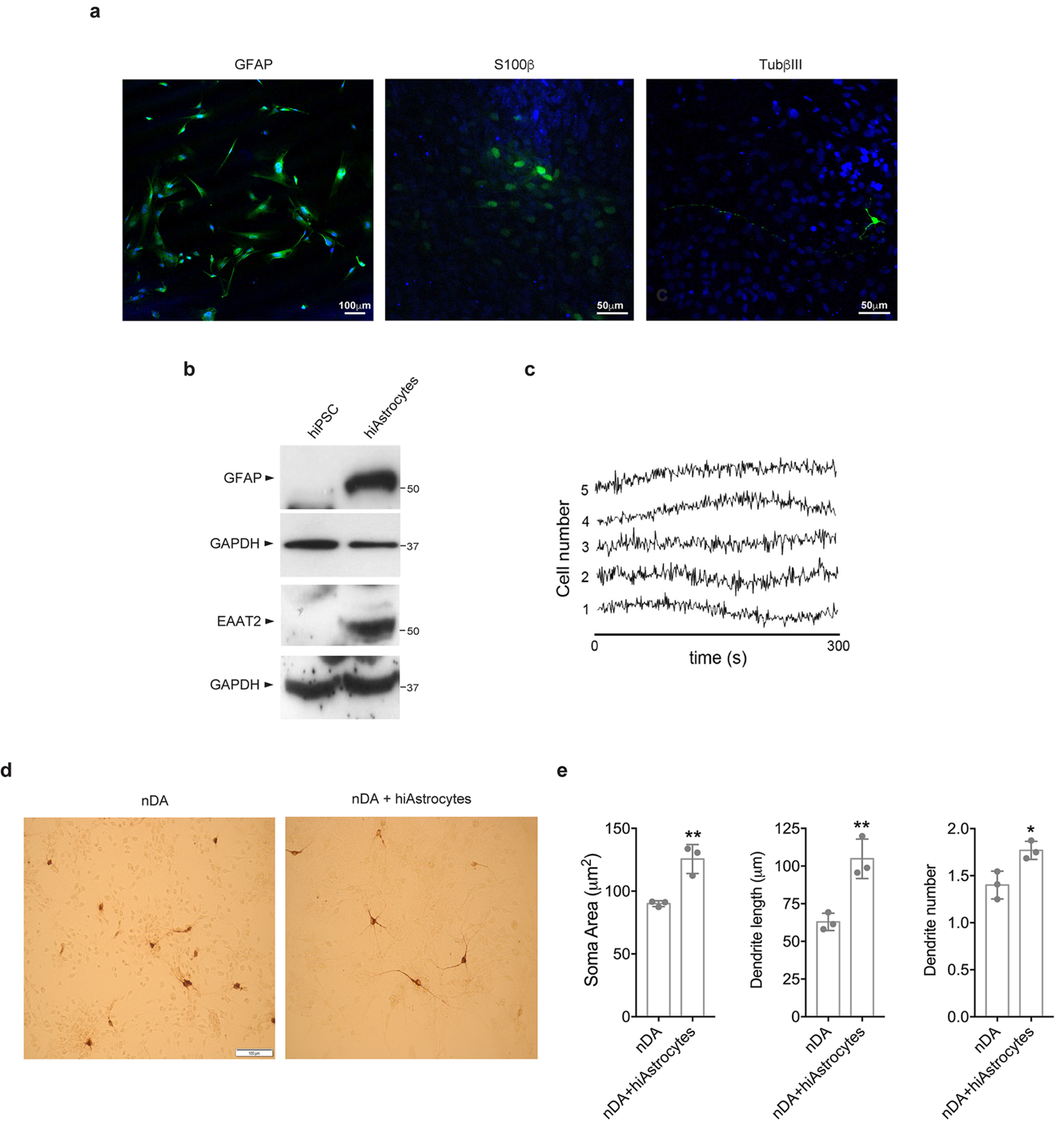


**Supplementary Figure 3. Generation and characterization of human iPSC-derived astrocytes. (a)** Maximum Intensity Z-projection confocal images of astrocyte marker GFAP and S100β and neuronal marker TubβIII in hiAstrocytes culture (25 days of differentiation). Scale bars 100 and 50 µm. **(b)** Cell lysates from hiPSC and hiAstrocytes (25 days of differentiation) were subjected to immunoblotting using astrocyte markers GFAP and EAAT2 antibodies; GAPDH has been used as loading control. **(c)** Graph representing functional Ca^2+^ waves of 5 single hiAstrocytes (25 days of differentiation). **(d)** Representative images of TH immunohistochemistry of murine dopaminergic neurons (nDA) cultured alone or co-cultured with hiAstrocytes for 7 days (33-40 days of differentiation). **(e)** Quantification of soma area, dendrite length and dendrite number of murine dopaminergic neurons (nDA) cultured alone or co-cultured with hiAstrocytes for 7 days (33-40 days of differentiation). At least sixty cells from three independent experiments were analyzed. Data are expressed as the mean ± SD; individual points represent each single experiment. Data were analyzed by unpaired t-test; soma area, ^**^p =0.0063; dendrite length, ^**^p =0.0072 and dendrite number, *p =0.022.
